# Recyclable Endogenous H_2_S Activation of Self‐Assembled Nanoprobe with Controllable Biodegradation for Synergistically Enhanced Colon Cancer‐Specific Therapy

**DOI:** 10.1002/advs.202203902

**Published:** 2022-09-30

**Authors:** Junqing Huang, Zhiming Deng, Shenghui Bi, Xingwang Wen, Songjun Zeng

**Affiliations:** ^1^ School of Physics and Electronics Key Laboratory of Low‐dimensional Quantum Structures and Quantum Control of the Ministry of Education Synergetic Innovation Center for Quantum Effects and Applications Key Laboratory for Matter Microstructure and Function of Hunan Province Hunan Normal University Changsha Hunan 410081 China

**Keywords:** controlled biodegradation, H_2_S‐activated self‐assembled nanoprobes, precise/sustainable cancer therapy, sustainable cyclic depletion of H_2_S/GSH

## Abstract

Excessive production of hydrogen sulfide (H_2_S) plays a crucial role in the progress of colon cancer. Construction of tumor‐specific H_2_S‐activated smart nanoplatform with controllable biodegradation is of great significance for precise and sustainable treatment of colon cancer. Herein, an endogenous H_2_S triggered Co‐doped polyoxometalate (POM‐Co) cluster with self‐adjustable size, controlled biodegradation, and sustainable cyclic depletion of H_2_S/glutathione (GSH) is designed for synergistic enhanced tumor‐specific photothermal and chemodynamic therapy. The designed POM‐Co nanocluster holds H_2_S responsive “turn‐on” photothermal property in colon cancer via self‐assembling to form large‐sized POM‐CoS, enhancing the accumulation at tumor sites. Furthermore, the formed POM‐CoS can gradually biodegrade, resulting in release of Co^2+^ and Mo^6+^ for Co(II)‐catalyzed •OH production and Russell mechanism‐enabled ^1^O_2_ generation with GSH consumption, respectively. More importantly, the degraded POM‐CoS is reactivated by endogenous H_2_S for recyclable and sustainable consumption of H_2_S and GSH, resulting in tumor‐specific photothermal/chemodynamic continuous therapy. Therefore, this study provides an opportunity of designing tumor microenvironment‐driven nanoprobes with controllable biodegradation for precise and sustainable anti‐tumor therapy.

## Graphical abstract

An endogenous H_2_S responsive “turn‐on” therapeutic Co‐doped polyoxometalate (POM‐Co) nanocluster with self‐adjustable size, controlled biodegradation, and sustainable cyclic depletion of H_2_S/glutathione (GSH) is designed for synergistic enhanced colon tumor‐specific photothermal and chemodynamic therapy.

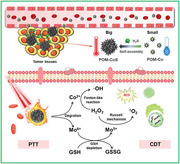

## 1 Introduction

Colon cancer is a malignant tumor that originates from the epithelial cells of the colonic mucosa.^[^ ^1^ ^]^ It is currently one of the three most common cancers in the world.^[^ ^2^, ^3^ ^]^ Many traditional treatment methods for colon cancer, including surgical resection, radiotherapy, and chemotherapy have been developed,^[^ ^4^, ^5^ ^]^ but these methods are often accompanied with various side effects, such as pain, fatigue, and drug resistance.^[^ ^6^, ^7^ ^]^ Thus, it is imperative to develop minimally invasive and highly effective treating method for colon cancer. Chemodynamic therapy (CDT) is a new minimally invasive treatment method, which utilizes Fenton or similar Fenton catalyst to convert hydrogen peroxide (H_2_O_2_) overexpressed in tumors into highly toxic hydroxyl radical (•OH) to selectively induce cell apoptosis and necrosis.^[^ ^8^, ^9^, ^10^, ^11^, ^12^, ^13^ ^]^ However, the efficacy of CDT was still limited by the complex tumor microenvironment (TME) of colon cancer. The TME of colon cancer not only has the same abundant glutathione (GSH) and H_2_O_2_, and low pH value as other tumors, but also has its unique high expression of endogenous hydrogen sulfide (H_2_S, 0.3–3.4 mM).^[^ ^14^, ^15^ ^]^ High expression of GSH and H_2_S could eliminate the strong oxidizing ability of •OH through their powerful reducing ability, greatly weakening the therapeutic effect of CDT.^[^ ^16^, ^17^, ^18^ ^]^ Notably, H_2_S can also promote colon cancer cell proliferation and angiogenesis around tumor tissues.^[^ ^19^, ^20^, ^21^ ^]^ Therefore, reshaping the TME of colon cancer via reducing H_2_S and GSH is extremely urgent to improve the efficiency of CDT in colon cancer. However, enhancing colon cancer CDT by effectively and completely removing endogenous H_2_S and GSH was rarely reported.

On the other hand, single CDT is not sufficient for anti‐tumor therapy. It is highly desirable to explore multifunction combinational therapy strategy for treatment of tumor. In recent years, photothermal therapy (PTT) is another new minimally invasive and controllable treatment method. It uses photothermal conversion materials to absorb laser energy and release heat to eliminate malignant tumors, which has attracted great attention and provided new opportunities for the treatment of colon cancer.^[^ ^22^, ^23^, ^24^, ^25^ ^]^ However, most photothermal agents tend to exhibit an always‐on hyperthermic effect and non‐specific therapy and biodistribution, resulting in inevitable damage to the normal tissue surrounding the tumor.^[^ ^26^, ^27^ ^]^ In addition, the long‐term retention of inorganic nanoparticles in the body possesses a great threat to health. Thereby, developing a new biodegradable probe to reduce heat‐induced non‐specific damage to normal tissue is of great significance to reduce the side effects during tumor therapy. It is well known that the size of nanomaterials is a key determinant of their mode of interaction with the biological environment and may fundamentally affect their performance in clinical settings.^[^ ^28^, ^29^ ^]^ From a clinical standpoint, large‐sized nanoparticles (>100 nm) can take advantage of the enhanced permeability and retention (EPR) effect, but due to the dense extracellular matrix and the increased fluid pressure between tissues, they cannot fully penetrate into the tumor tissues, resulting in poor therapeutic effects.^[^ ^30^, ^31^ ^]^ Small‐sized nanoparticles exhibit better tumor penetration ability and spread more uniformly in tumor tissues. But because it is easily excreted by the body, resulting in insufficient tumor accumulation.^[^ ^32^, ^33^, ^34^ ^]^ Therefore, design of tumor specific H_2_S‐activated nanoprobe with self‐adjustable size, controllable biodegradation and synergistically enhanced CDT/PTT is highly demanded for precise therapy of colon cancer.

**Scheme 1:**
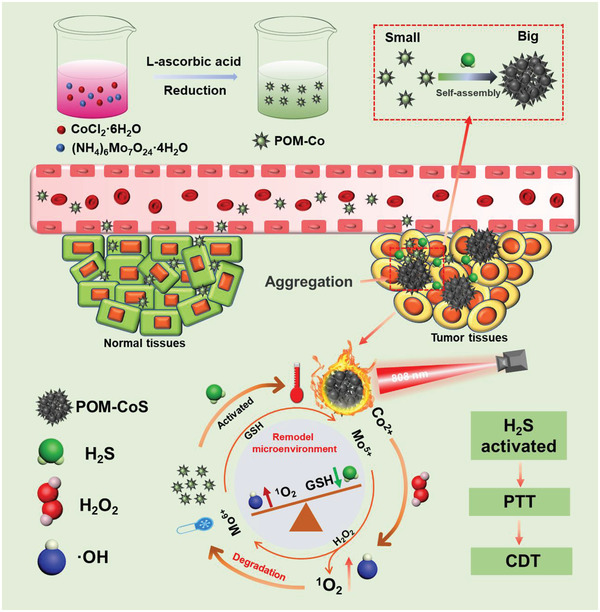
Schematic illustration of the H_2_S‐activated self‐aggregation of POM‐Co in vivo for synergistically enhanced PTT/CDT of colon tumor with sustainable H_2_S and GSH consumption.

Herein, the endogenous H_2_S responsive “turn‐on” therapeutic Co‐doped molybdenum‐based polyoxometalate cluster (denoted as POM‐Co) is designed for targeted therapy of H_2_S‐rich colon cancer (**Scheme** **1**). The obtained POM‐Co clusters maintained a small size in normal tissues and have barely near‐infrared (NIR) absorption. However, in colon cancer, POM‐Co clusters rapidly react with H_2_S to form self‐assembled POM‐CoS with large size and exhibit strong NIR absorption, thereby enhancing the accumulation of nanoprobe in tumor and improving the specificity of photothermal therapy. Furthermore, the released Co^2+^ and Mo^6+^ from the biodegradation of POM‐CoS can induce •OH and ^1^O_2_ production with GSH consumption, respectively. Notably, the degraded POM‐CoS can be re‐activated by endogenous H_2_S, leading to continuous and cyclic consumption of H_2_S and GSH. Thus, the designed POM‐Co agents could not only remodel the endogenous H_2_S and GSH microenvironment in colon cancer, but also synergistically enhance CDT and PTT.

## 2 Results and Discussion

**Figure 1:**
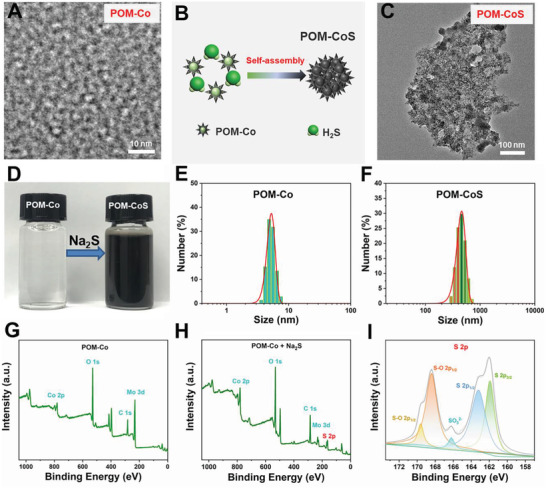
A) TEM image of POM‐Co clusters. B) Self‐aggregation mechanism diagram of POM‐Co after adding H_2_S. C) TEM image of POM‐CoS clusters. D) A photograph of POM‐Co clusters in the presence of Na_2_S. E) Dynamic light scattering measurements of the size of POM‐Co clusters. F) Dynamic light scattering measurements of the size of POM‐CoS clusters. G) Full XPS of POM‐Co clusters. H) Full XPS of POM‐Co clusters after adding Na_2_S. I) High‐resolution XPS of S 2p.

Due to the limitation of CDT by overexpressed H_2_S in colon cancer, and the non‐specificity of most photothermal agents, we designed an endogenous H_2_S triggered POM‐Co clusters with “turn‐on” photothermal properties, sustainable H_2_S consumption and biodegradation for highly specific and synergistically enhanced PTT/CDT of colorectal tumor (Scheme 1). As demonstrated in the transmission electron microscope (TEM) images (**Figure** **1**A,E), the designed POM‐Co clusters have ultra‐small diameters (≈5.28 nm) in water, along with zeta potential of –9.09 mV (Figure S1, Supporting Information). Then, Na_2_S was used to simulate endogenous H_2_S for investigating the reaction of POM‐Co clusters with endogenous H_2_S (Figure 1B). After the addition of Na_2_S, POM‐Co solution changed from colorless to black immediately (Figure 1D), indicating that Na_2_S can react with POM‐Co clusters quickly. At the same time, small size POM‐Co clusters could rapidly self‐assemble into large size (>400 nm) to form POM‐CoS clusters (Figure 1C,F) via reacting with Na_2_S. In order to further elucidate the composition and valence states of POM‐Co and POM‐CoS clusters, the energy dispersive X‐ray spectroscopy (EDS) and the X‐ray photoelectron spectroscopy (XPS) analyses were performed. As demonstrated in Figure S2 (Supporting Information) and Figure 1G, the Mo 3d, Co 2p, O 1s signals were clearly observed from the EDS and XPS results, indicating that Co was successfully incorporated into the molybdenum polyoxomethoate clusters, and the molar ratio of Mo and Co is about 5.94:3.62 (Table S1, Supporting Information). The Mo 3d exhibits two main fitting peaks at 235.8 eV (Mo 3d3/2) and 232.7 eV (Mo 3d5/2),^[^ ^35^, ^36^, ^37^, ^38^ ^]^ which can be assigned to Mo^6+^. And the obvious peaks at 797.6 eV (Co 2p1/2) and 781.8 eV (Co 2p3/2) are assigned to the Co^2+^ (Figure S3, Supporting Information).^[^ ^39^, ^40^ ^]^ In addition, after the reaction of POM‐Co with Na_2_S, the obtained sample showed S 2p signal, indicating the formation of POM‐CoS (Figure 1H). Moreover, the peaks of S element at 162.0 and 163.2 eV correspond to S 2p3/2 and S 2p1/2, respectively, indicating the formation of sulfide (Figure S4, Supporting Information and Figure 1I).^[^ ^41^ ^]^ Besides, the peaks of S element at 168.5 and 169.6 eV can be attributed to the S—O bonds formed by the surface oxidation of POM‐CoS.^[^ ^42^ ^]^ These characterizations further confirmed the phase and structure conversion of POM‐Co to POM‐CoS.

**Figure 2:**
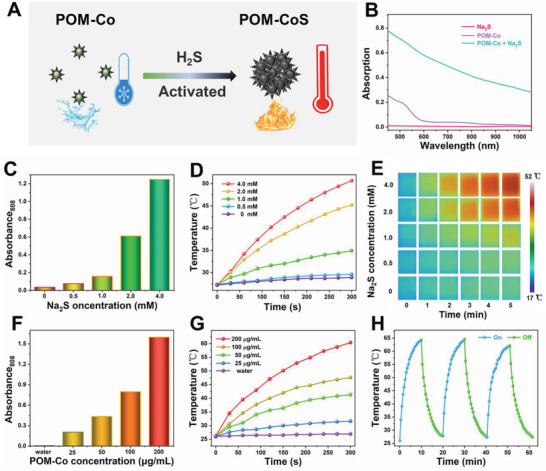
A) Mechanism of H_2_S‐activated photothermal property. B) The UV–vis absorption spectra of Na_2_S, POM‐Co and POM‐CoS. C) Absorbance at 808 nm of POM‐Co clusters reacted with different Na_2_S concentrations. D) Representative temperature variation curves and E) corresponding thermal images of the aqueous dispersion of POM‐Co clusters at various concentrations (0, 0.5, 1, 2, and 4 mMm) with addition of Na_2_S under 808 nm laser irradiation. F) Absorbance at 808 nm of Na_2_S reacted with different POM‐Co clusters concentrations. G) Corresponding temperature change profile versus time of POM‐Co clusters (0–200 µg mL^−1^) reacted with 4 mM Na_2_S under irradiation with an 808 nm laser. H) Photothermal stability of POM‐CoS clusters (three ON/OFF cycles).

To reveal the H_2_S‐triggered “turn‐on” photothermal properties of POM‐Co, its absorption spectra were first measured. As illustrated in **Figure** **2**A,B, the POM‐Co solution exhibits very weak absorption in the NIR region. After introducing Na_2_S, the solution held strong absorption at 808 nm, leading to the activated photothermal nature for highly specific PTT of colon cancer with overexpressed H_2_S. In order to further investigate the H_2_S triggered thermal performance, the POM‐Co clusters were incubated with different concentrations of Na_2_S. As shown in Figure 2C, with the increase of Na_2_S concentration, the absorbance of POM‐Co solution at 808 nm gradually increased. In order to further reveal the photothermal efficiency, we tested the corresponding temperature curve and thermal images. As shown in Figure 2D,E, the temperature of pure POM‐Co solution increased by only 1.7 °C, while the temperature increased by 2.3–23.5 °C with the addition of Na_2_S from 0.5 to 4 mM. The similar changes of absorbance and temperature were observed when adding different concentrations of POM‐Co aqueous solution to Na_2_S (Figure 2F,G and Figure S5, Supporting Information), indicating the efficient Na_2_S activated NIR photothermal nature. The photothermal conversion efficiency of POM‐CoS was further determined to be 40.6% (Figure S6, Supporting Information) by the previously reported method.^[^ ^25^ ^]^ After 3 cycles of on/off (Figure 2H), the maximum temperature of the POM‐CoS solution remained basically unchanged, indicating the good photothermal stability of POM‐CoS. At present, various nanomaterials have been used in PTT of tumors, but most drugs exhibit “always on” PTT function, which may increase damage to normal tissues. In contrast, the designed POM‐Co clusters can effectively avoid this side effect and realize highly specific PTT in the tumor region, providing precise and specific treatment for colon cancer.

**Figure 3:**
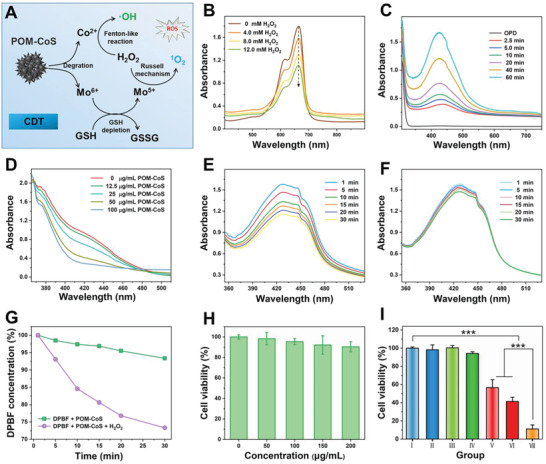
A) Mechanism diagram of Co(II) Fenton‐like catalytic and Russell mechanism of Mo for generating •OH and ^1^O_2_. B) The absorption curve of MB. C) Time‐dependent absorption spectra of OPD after the POM‐CoS mediated Fenton‐like reaction. D) GSH depletion under the reduction of different concentrations of POM‐CoS (12.5, 25, 50, 100 µg mL^−1^). E,F) Time‐dependent DPBF absorption curves of POM‐CoS reacting with or without H_2_O_2_, respectively and G) the corresponding relative absorption at 419 nm of DPBF. H) Cytotoxicity of POM‐Co clusters with different concentrations. I) The cell viability of CT26 cells with different treatments. Data are presented as mean ± SD. *P* values: ***p < 0.001.

It is noteworthy that POM‐CoS not only has excellent photothermal conversion ability, but also possesses catalytic ability (**Figure** **3**A). In recent years, cobalt chalcogenide compounds have broad application prospects in the field of CDT due to their unique properties similar to Fenton reaction. In order to evaluate the Co(II)‐mediated Fenton‐like reaction, the absorption spectrum of methylene blue (MB) at 664 nm was used to detect the generation of •OH. As shown in Figure 3B, the characteristic peak of MB gradually decreases with increasing H_2_O_2_, mainly because the Co^2+^ ions could react with H_2_O_2_ to generate •OH (Co^2+^ + H_2_O_2_ → Co^3+^ + •OH). Simultaneously, Co^3+^ can also oxidize H_2_O_2_ to produce Co^2+^ (Co^3+^ + H_2_O_2_ → Co^2+^ + H_2_O + O_2_).^[^ ^43^, ^44^, ^45^ ^]^ The o‐phenylenediamine (OPD) probes were further used to evaluate the production of •OH. After POM‐CoS and OPD were incubated, OPD gradually changed from colorless to yellow with the addition of H_2_O_2_, and the absorbance at 425 nm (Figure 3C) gradually increased. This is mainly ascribed to the conversion of OPD to diaminobenazine (DAP) via Fenton reaction, further proving the generation of •OH.^[^ ^36^ ^]^ In addition, using 5,5‐dimethyl‐1‐pyrroline *N*‐oxide (DMPO) as the capture probe, electron spin resonance (ESR) spectroscopy experiments were performed to further confirm the generation of •OH radicals. Compared with the pure H_2_O_2_ solution, a typical 1:2:2:1 ESR peak appeared when POM‐CoS was added to H_2_O_2_ solution, indicating the production of •OH (Figure S7, Supporting Information). It is well known that GSH is an intracellular antioxidant, which can remove highly active •OH and ^1^O_2_. Therefore, in order to improve the efficiency of CDT, it is necessary to eliminate GSH in tumor cells. Fortunately, the endogenous GSH can reduce the unique components of Mo^6+^ in POM‐CoS, thereby consuming GSH. In order to study the consumption of GSH by POM‐CoS, 5,5′‐dithiobis(2‐nitrobenzoic acid) (DTNB)^[^ ^39^ ^]^ was used as a probe for evaluation. As the concentration of POM‐CoS increases, the absorption peak of DTNB at 412 nm decreases (Figure 3D), verifying the efficient consumption of GSH. At the same time, the polyoxometalate reduced to Mo^5+^ by GSH will react with H_2_O_2_ to form a tetraoxide, and then decompose to ^1^O_2_ (Figure S8, Supporting Information),^[^ ^38^ ^]^ which is further confirmed by the presence of the reduction peak of Mo^5+^ at 226.8 eV in the XPS spectrum (Figure S9, Supporting Information). And 1,3‐Diphenylisobenzofuran (DPBF) was used to verify the production of ^1^O_2_. As depicted in Figure 3E–G, the absorption intensity of POM‐CoS treated by GSH maintained unchanged after mixing with DPBF, while after adding H_2_O_2_, the absorption intensity decreased sharply, implying efficient ^1^O_2_ production. Subsequently, the generation of ^1^O_2_ was further verified by ESR using 2,2,6,6‐tetramethylpiperidine (TEMP) as the ^1^O_2_ scavenger. As shown in Figure S10 (Supporting Information), compared with the blank control group, the 1:1:1 triplet signal peak of ^1^O_2_ in the POM‐CoS treated by GSH and H_2_O_2_ mixture group was significantly enhanced. Excellent biosafety and biocompatibility are the basic prerequisites for the application of POM‐Co in vivo. Therefore, we performed the hemolysis test of POM‐Co nanoclusters. As shown in Figure S11 (Supporting Information), the POM‐Co nanoprobes exhibited good blood compatibility. Next, the cytotoxicity of POM‐Co was investigated using the standard CCK8 method. As shown in Figure 3H, when treating CT26 cells with POM‐Co (200 µg mL^−1^) for 24 h, the cell viability was not significantly inhibited, which proved its good biocompatibility. Subsequently, the in vitro therapeutic capacity of POM‐Co was evaluated. CT26 cancer cells in different conditions (I: PBS, II: NIR, III: Na_2_S + H_2_O_2_, IV: POM‐Co, V: POM‐Co + Na_2_S + H_2_O_2_, VI: POM‐Co + Na_2_S + NIR, VII: POM‐Co + Na_2_S + H_2_O_2_ + NIR) were treated. As demonstrated in Figure 3I, compared with the control group, no obvious cytotoxicity was observed in the NIR, Na_2_S + H_2_O_2_ and POM‐Co groups, while POM‐Co + Na_2_S + H_2_O_2_, POM‐Co + Na_2_S + NIR, and POM‐Co + Na_2_S + H_2_O_2_ + NIR groups showed obvious therapeutic effect. And, in POM‐Co + Na_2_S + H_2_O_2_ and POM‐Co + Na_2_S + NIR groups, the cell viability dropped to 56% and 42%, respectively, because of the generation of reactive oxygen species by POM‐CoS in the presence of H_2_O_2_ and H_2_S‐activated PTT. Notably, the cells in the POM‐Co + Na_2_S + H_2_O_2_ + NIR group had the highest cell lethality, which was mainly due to the synergistic effect of CDT and PTT. These results suggest that the designed POM‐Co can be used as an ideal H_2_S‐responsive “turn‐on” therapeutic nanoprobe for antitumor therapy.

**Figure 4:**
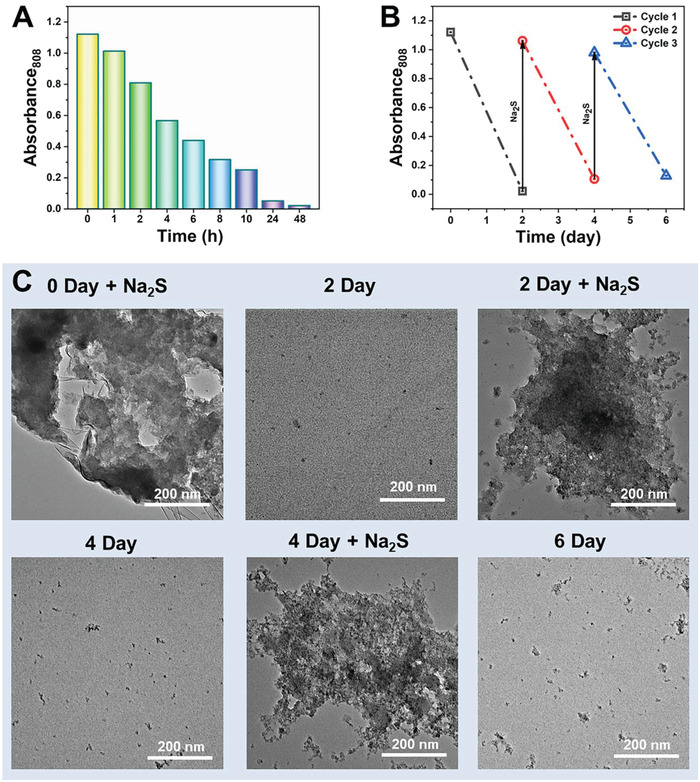
A) Absorbance at 808 nm of POM‐CoS clusters in deionized water at different time points. B) The absorbance at 808 nm after reacting with Na_2_S and degradation. C) TEM images of POM‐Co reacted with Na_2_S and degradation over time.

Biodegradation and recyclable behavior of nanomaterials are critical for their biomedical applications. in vitro biodegradation behavior of POM‐CoS in aqueous solution was investigated. As shown in Figure S12 (Supporting Information) and **Figure** **4**A, with the prolongation of immersion time, the POM‐CoS suspension gradually became transparent from black, and the corresponding UV–vis absorption value at 808 nm also decreased significantly. In order to further understand the degradation mechanism of POM‐CoS, the morphology of POM‐CoS was studied by TEM. As shown in Figure S13 (Supporting Information), POM‐CoS gradually changed from a large cluster structure to a small cluster over time (0–48 h), and only some small particles could be observed after two days. In order to understand the degradation mechanism of POM‐CoS, XPS was used to evaluate the change of valence state and composition of POM‐CoS after the degradation. According to the XPS spectrum of S, compared with POM‐CoS before degradation, the signal of S 2p was attenuated, while the signal of SO_4_^2−^ was enhanced (Figure S14, Supporting Information). It can be seen from the above results that the degradation mechanism may be due to the transfer of POM‐CoS to soluble sulfate. In recent years, a series of biodegradable nano‐drugs have been studied, but little attention has been paid to recyclable nano‐drugs. In order to study the recyclability of POM‐Co, the reaction of POM‐Co and Na_2_S and its degradation were repeated three times. It can be seen that the value of the absorbance values at 808 nm (Figure 4B) of each reaction cycle is basic same. At the same time, the photothermal performance of POM‐Co reacted with Na_2_S in each cycle was studied, and it was found that after three activations and degradations, the photothermal conversion efficiency could still reach 39.01% (Figure S15, Supporting Information), which was almost consistent with the photothermal conversion efficiency (40.6%) of the first activation. Therefore, POM‐Co can be used as a recyclable photothermal agent. TEM also confirmed the recyclability of the designed nanoclusters (Figure 4C).

**Figure 5:**
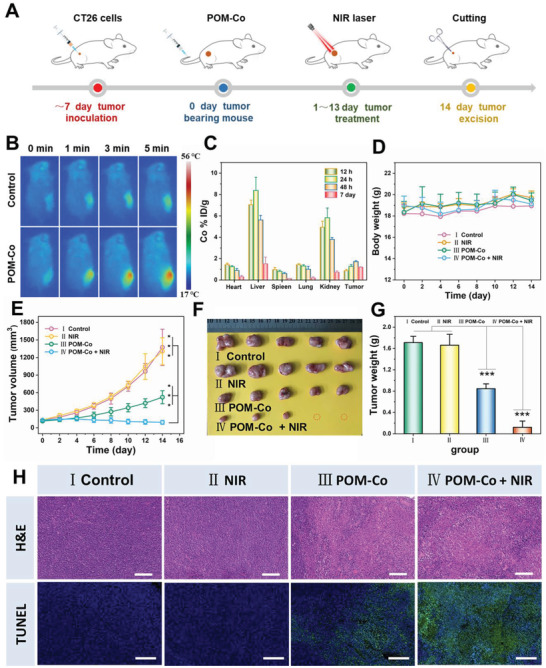
A) Schematic diagram of in vivo treatment procedures. B) Thermal imaging of tumor‐bearing mice injected with POM‐Co or PBS under 808 nm laser irradiation. C) Biodistribution of the designed probe in tumor and major tissues (heart, liver, spleen, lung, and kidney) at 12, 24, 48 h, and 7 day after intravenous injection of POM‐Co. D) Variation of body weight in the different treatment groups over 14 days. E) Variation of tumor volume of mice after different treatments. F) Photograph of tumor removal from different groups of mice after 14 days of treatment. G) Weight of dissected tumor of each group. H) Representative images of H&E and TUNEL staining of tumor tissues from different groups. Scale bar = 200 µm. Data are presented as mean ± SD. *P* values: ***p < 0.001.

Compared with other types of cancer, overexpressed H_2_S offers the possibility of precise treatment for colon cancer. To demonstrate this hypothesis, we established CT26 tumor‐bearing mice to evaluate the in vivo therapeutic efficacy of H_2_S‐activated POM‐Co nanoclusters. Afterward, the tumor‐bearing mice were randomly divided into four groups (**Figure** **5**A): I) control; II) NIR; III) POM‐Co; IV) POM‐Co + NIR. In order to verify the “turn‐on” PTT triggered by H_2_S, thermal imaging of tumor‐bearing mice was first performed under laser irradiation (Figure 5B and Figure S16, Supporting Information). Compared with the mice injected with PBS, the tumor site of the mice injected with POM‐Co produced significant heat, and the terminal temperature reached 48 °C, indicating the successful endogenous H_2_S triggered structure transformation of POM‐Co into POM‐CoS and activation of photothermal property. The biodistribution in major organs and tumors was also quantitatively analyzed by inductively coupled plasma mass spectroscopy (ICP‐MS) after intravenous injection of POM‐Co for 12 h, 24 h, 48 h, and 7 days. As shown in Figure 5C, the specific uptake of POM‐Co in tumors continued to increase and remained accumulation in tumors up to 7 days after injection, possibly due to the H_2_S‐driven self‐assembly of POM‐Co in tumors. Notably, it is expected that the H_2_S‐triggered aggregation state can degrade to an ultra‐small state after tumor cure. As demonstrated, most POM‐Co nanoclusters were removed from major organs, indicating that the prepared POM‐Co nanoclusters have good biosafety in vivo. The circulation of POM‐Co nanoclusters in the blood was studied, and the half‐life of POM‐Co nanoclusters was calculated to be about 1.63 h (Figure S17, Supporting Information), indicating the good in vivo circulation behavior of the nanocluster.

The changes in body weight and tumor volume of the mice in each group during the treatment period were further recorded. The weight curve showed that the weight of the experimental group did not change significantly (Figure 5D) compared with the control group, indicating the high biocompatibility. Compared with the control group, tumor growth was partially inhibited in the POM‐Co group (Figure 5E**–**G), which was ascribed to the endogenous overexpressed H_2_O_2_‐triggered CDT to generate toxic •OH and ^1^O_2_. Notably, the POM‐Co + NIR group showed better treatment outcomes than the POM‐Co group due to the combination of CDT and PTT. H&E and terminal deoxynucleotidyl transferase dUTP nick end labeling (TUNEL) staining tests were used to analyze tumor sections from different treatment groups in order to further reveal their antitumor effects. As shown in Figure 5H, more serious tumor damage and necrosis were detected in the POM‐Co + NIR group compared with the POM‐Co group. However, no obvious tumor cell apoptosis and necrosis were found in the control and NIR groups. To further assess the biosafety of POM‐Co nanoclusters, we also performed the histological analysis of major organs (heart, liver, spleen, lung, and kidney) of normal mice intravenously injected with the probe for 7, 15, and 30 days (Figure S18, Supporting Information), illustrating its excellent biocompatibility. In addition, we performed blood biochemical analyses, including aspartate aminotransferase (AST), alanine aminotransferase (ALT), blood urea nitrogen (BUN), and creatinine. As shown in Figure S19 (Supporting Information), there is no statistical difference between the test and control group, further confirming its high biocompatibility. These results demonstrate that TME‐driven POM‐Co is a precise, efficient, and biocompatible multifunctional nanoprobe for sustainable and synergistic tumor therapy.

## 3 Conclusion

In summary, we explored a smart POM‐Co nanoprobe self‐powered by H_2_S for precisely targeted treatment of colorectal cancer. POM‐Co was rapidly self‐aggregated into the large sized POM‐CoS nanoclusters with “turn‐on” photothermal properties by the endogenous H_2_S overexpressed in colon cancer, which facilitated the aggregation and retention of POM‐Co in colon cancer. The activated POM‐CoS clusters could not only trigger the conversion of endogenous H_2_O_2_ into •OH and ^1^O_2_, but also reduce the intracellular GSH level, thereby breaking the redox balance inside the tumor and amplifying the CDT effect. The synergistic effect of PTT and CDT could induce the effective death of cancer cells in vitro, completely remove tumors in vivo, and has no side effects on normal tissues. Interestingly, the designed nanodrug presents cyclic degradation and aggregation properties, uninterruptedly scavenging H_2_S/GSH and generating toxic •OH and ^1^O_2_. More importantly, after therapy, the aggregated POM‐CoS can be degraded into small particles in vivo and then removed from the body via the renal excretion pathway. Therefore, this study proposes a multifunctional nanoplatform for recyclable pathological stimuli‐responsive combination therapy, providing a promising strategy to construct smart responsive theranostic agent for tumor‐specific therapy.

## Conflict of Interest

The authors declare no conflict of interest.

## Supporting information

Supporting Information

## Data Availability

Research data are not shared.
