# Supplementary material for: Recyclable Endogenous H2S Activation of Self‐Assembled Nanoprobe with Controllable Biodegradation for Synergistically Enhanced Colon Cancer‐Specific Therapy
Source: Adv Sci (Weinh). 2022 Sep 30;9(31):2203902. doi: 10.1002/advs.202203902 (PMC9631061; doi:10.1002/advs.202203902)
Supplement: Supplementary file 1 — Supporting Information [file ADVS-9-2203902-s001.pdf]

ADVANCED SCIENCE
Open Access

# Supporting Information

for *Adv. Sci.*, DOI 10.1002/advs.202203902

Recyclable Endogenous $H_2S$ Activation of Self-Assembled Nanoprobe with Controllable Biodegradation for Synergistically Enhanced Colon Cancer-Specific Therapy

*Junqing Huang, Zhiming Deng, Shenghui Bi, Xingwang Wen and Songjun Zeng**

# Supplement Information

## Recyclable Endogenous $H_2S$ Activation of Self-Assembled Nanoprobe with Controllable Biodegradation for Synergistically Enhanced Colon Cancer-Specific Therapy

*Junqing Huang, Zhiming Deng, Shenghui Bi, Xingwang Wen, Songjun Zeng**

School of Physics and Electronics, Key Laboratory of Low-dimensional Quantum Structures and Quantum Control of the Ministry of Education, Synergetic Innovation Center for Quantum Effects and Applications, Key Laboratory for Matter Microstructure and Function of Hunan Province, Hunan Normal University, Changsha, Hunan 410081, China.

*Email:* songjunz@hunnu.edu.cn

# MATERIALS AND METHODS

## Materials

$CoCl_2 \cdot 6H_2O$, $(NH_4)_6Mo_7O_{24} \cdot 4H_2O$, L-ascorbic acid, 5,5-dimethyl-1-pyrroline *N*-oxide (DMPO), 2,2,6,6-tetramethylpiperidine (TEMP), 1,3-Diphenylisobenzofuran (DPBF), GSH, 5,5′-dithiobis(2-nitrobenzoic acid) (DTNB), o-phenylenediamine (OPD) and methylene blue (MB) were all obtained from Sinopharm Chemical Reagent Co. Ltd. All chemicals were used directly without further purification.

## Synthesis of POM-Co cluster

The POM cluster was simply synthesized through the one-pot method modified as previous report.[S1] At first, $(NH_4)6Mo_7O_{24} \cdot 4H_2O$ (0.4414 g) was dissolved in 10 mL ultrapure water and stirred for 0.5 h at room temperature. Subsequently, 5 mL solution of $CoCl_2 \cdot 6H_2O$ (35 mg/mL) was added and stirred for another 0.5 h. At last, 2 mL solution of L-ascorbic acid (100 mg/mL) was added dropwisely into the system under stirring. After stirring at room temperature for 2 h, 80 mL ethanol was added to precipitate. Finally, the samples were centrifuged and washed with water and ethanol for three times.

## Characterization

The morphology, structure and elemental compositions of the samples were characterized by transmission electron microscopy (TEM, FEI Tecnai F20) operated at 200 kV. The elements in the sample were further measured by the energy dispersive X-ray spectroscopy (EDS) and X-ray photoelectron spectroscopy (XPS, Thermo Fisher Scientific Escalab 250Xi). The UV-Vis adsorption spectra were recorded using the absorption spectrometer (Lambda750, Perkin-Elmer) at room temperature. The thermal imaging was recorded by Fluke Ti95 infrared camera.

## Measurement of photothermal performance and stability *in vitro*

The mixed solution of different concentrations of POM-Co (0, 25, 50, 100 and 200 μg/mL) and $Na_2S$, and the mixed solution of different concentrations of $Na_2S$ (0, 0.5, 1, 2 and 4 mM) and POM-Co were irradiated with an 808 nm laser (1 $W/cm^2$). Thermal infrared camera (Ti95, Fluke, USA) was used to record the temperature changes and real-time thermal images at the designed time intervals. The photothermal stability test was conducted by irradiating POM-CoS aqueous solution with 808 nm laser and then cooling it for three times. The photothermal conversion efficiency ($\eta$) of POM-CoS was calculated by the previous method.[S2,S3]

**The assay of •OH production**

MB (10 μg/mL), POM-CoS (100 μg/mL) and $H_2O_2$ with different concentrations were mixed and reacted for 10 min. MB degradation is detected with characteristic absorption at 665 nm. POM-Co was added in the solution containing OPD (1 mM) and $H_2O_2$ (10 mM). The •OH generation was monitored by the absorbance change at 452 nm.[S1] For electron spin resonance (ESR) assay, POM-CoS, $H_2O_2$ and DMPO were mixed and reacted for 10 min. EPR spectra were recorded.

**The assay of GSH consumption and $^1O_2$ production**

GSH (1 mM) was mixed with POM-CoS (12.5, 25, 50 and 100 μg/mL) for 2 h, and DTNB (0.5 mM) was added. The degradation of GSH was monitored by the absorbance change at 412 nm. The mixture of GSH (10 mM) and POM-CoS (100 μg/mL) was reacted for 12 h. DPBF (20 μg/mL) and 200 μL $H_2O_2$ (10 mM) were added into 400 μL of the mixture. The absorbance at 419 nm was monitored by absorption spectrometer. For ESR assay, the trapping agent TEMP of $^1O_2$ was added for 10 min, and then the EPR spectrum was recorded.

***In vitro* cell experiment**

The biocompatibility of POM-Co was evaluated by standard CCK8 assay. CT26 cells were inoculated in 96-well plates for 24 h, and then incubated with different concentrations of POM-Co for 24 h. Finally, the cells were washed with PBS and added with culture medium

and CCK8. The cells were further cultured at 37 °C for 2 h. The cytotoxicity was assessed by testing the absorbance at 450 nm via CCK8 assay.

**Tumor models**

$8 \times 10^6$ CT26 cancer cells were subcutaneously injected into BALB/C mice, after further cultured about four weeks, the tumor-bearing mouse models were obtained for *in vivo* synergistic antitumor therapy experiments.

***In vivo* pharmacokinetic and biodistribution assays**

For blood circulation assay, 15 μL blood sample was collected at different time points (5 min, 30 min, 1 h, 2 h, 4 h, 8 h, 24 h) after intravenous injection. And the Co content in blood was determined by inductively coupled plasma mass spectrometry (ICP-MS). Similarly, major organs (heart, liver, spleen, lung and kidney) and tumor (n=3) were harvested at time points of 12 h, 24 h, 48 h and 7 day for biodistribution assessment.

***In vivo* synergistic antitumor therapy**

The tumor-bearing BALB/C mice (CT26 cancer cells) were randomly divided into four groups (n=5 per group): Group I-only PBS group; Group II-solo 808 nm laser irradiation; Group III-POM-Co (150 μL, 2 mg/mL, single CDT group); Group IV-POM-Co solution (150 μL, 2 mg/mL) and 808 nm laser irradiation (PTT + CDT group). Mice from Group II and Group IV were irradiated with 808 nm laser at a power density of 1.5 $W/cm^2$ for 5 min at 24 h injection. The temperature change during the treatment was monitored by using a thermal camera. The tumor sites were irradiated 5 min by using the 808 nm laser every two days. The mice weights and tumor sizes were measured every 2 days.

**Tumor biopsies**

For hematoxylin and eosin (H&E) and Terminal deoxynucleotidyl transferase dUTP nick end labeling (TUNEL) staining, the tumors of CT26 tumor-bearing mice after different treatments were dissected. And then the tumor slices were stained with H&E and TUNEL for histological test.

**Blood biochemical analysis.**

For *in vivo* safety evaluation, the Kunming mice were injected with POM nanoprobes (3 mg/mL) for 14 days and control group without treatment. The blood samples were then collected for further biochemistry tests. And the biomarkers of alanine transferase (ALT), aspartate transaminase (AST), blood urea nitrogen (BUN) and creatinine were measured.

**Histopathological examination**

To evaluate the *in vivo* toxicity, the main organs including heart, liver, spleen, lung and kidney from untreated and treated normal mice (intravenous injection of POM-Co solution) for 7, 15 and 30 days were also collected for H&E staining.

**Statistical analysis**

The results of statistical analysis were presented as mean ± SD. Statistical significance was calculated by one-way ANOVA analysis. The statistical significance was defined as $*p < 0.05$; $**p < 0.01$; $***p < 0.001$.

## Supplement Reference

S1. Y. H. Shi, J. J. Zhang, H. Huang, C. Y. Cao, J. J. Yin, W. J. Xu, W. J. Wang, X. J. Song, Y. W. Zhang, X. C. Dong, *Adv. Healthcare Mater.* **2020**, *9*, 2000005.

S2. L. An, X. D. Wang, X. C. Rui, J. M. Lin, H. Yang, Q. W. Tian, C. Tao, S. P. Yang, *Angew. Chem., Int. Ed.* **2018**, *57*, 15782

S3. C. Tao, L. An, J. M. Lin, Q. W. Tian, S. P. Yang, *Small* **2019**, *15*, 1903473.

## Supplementary Figures

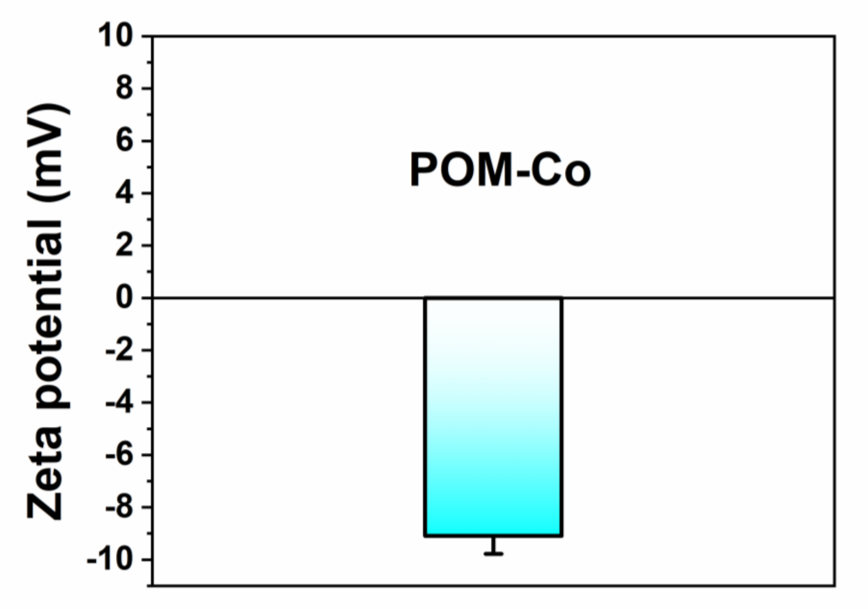


**Figure S1.** Zeta potential of POM-Co clusters.

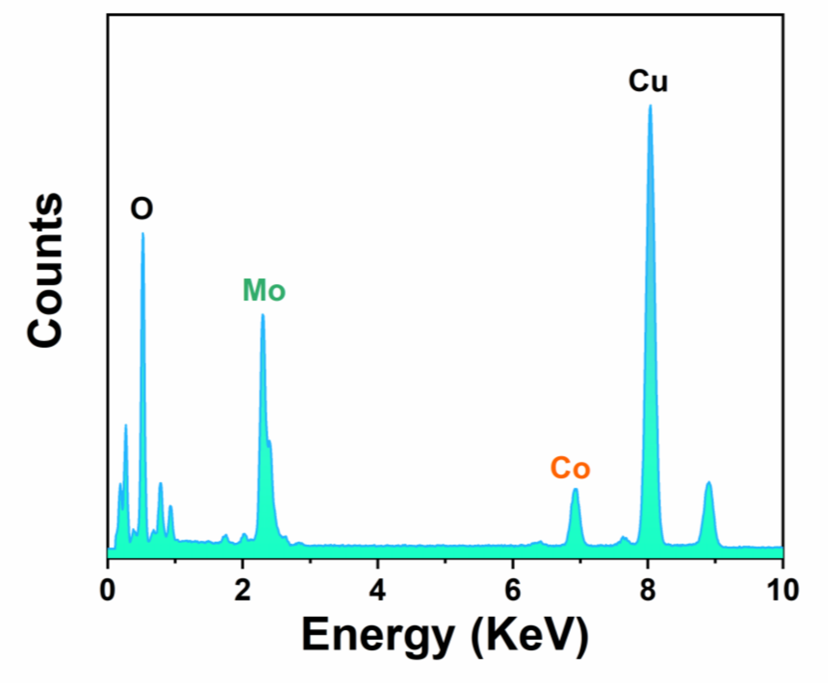


**Figure S2.** EDS pattern of POM-Co clusters.

**Table S1.** Element composition of POM-Co clusters from EDS analyses.

| Element | Weight % | Atomic % |
| --- | --- | --- |
| C | 8.00 | 24.10 |
| N | 0.64 | 1.66 |
| O | 14.77 | 33.40 |
| Co | 5.90 | 3.62 |
| Cu | 54.91 | 31.26 |
| Mo | 15.75 | 5.94 |

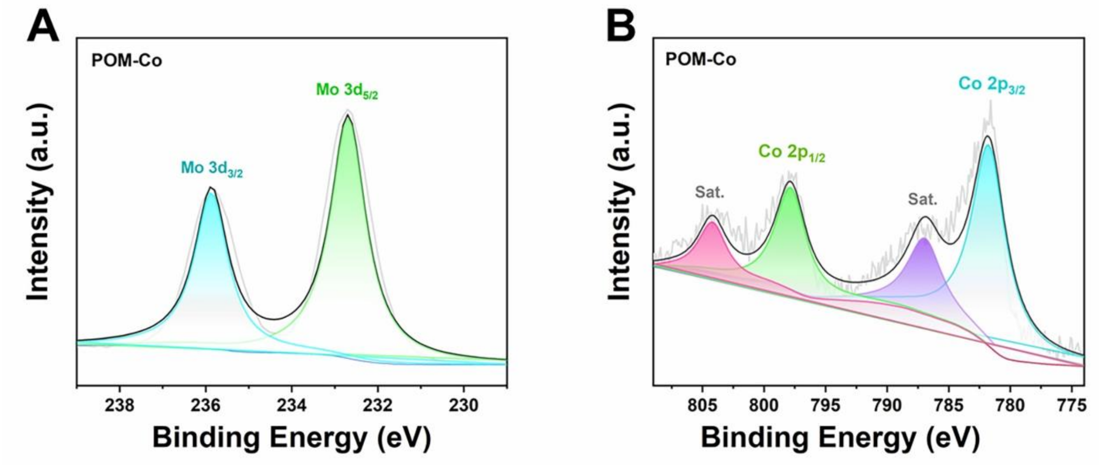


**Figure S3.** High resolution XPS spectra of (A) Mo and (B) Co of POM-Co clusters.

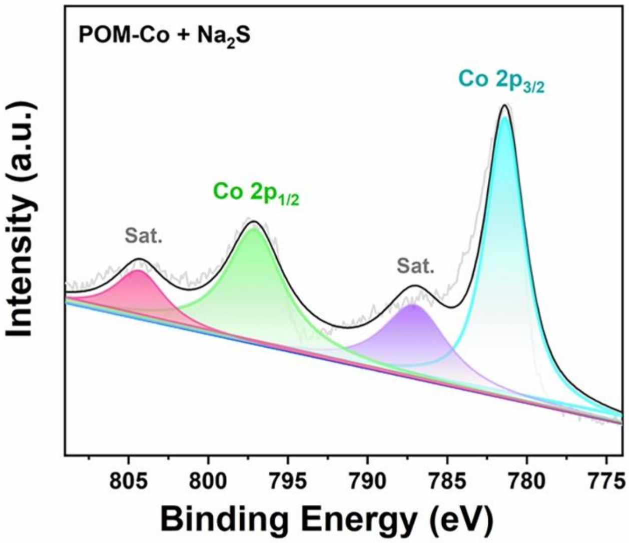


**Figure S4.** High resolution XPS spectrum of Co in POM-CoS clusters.

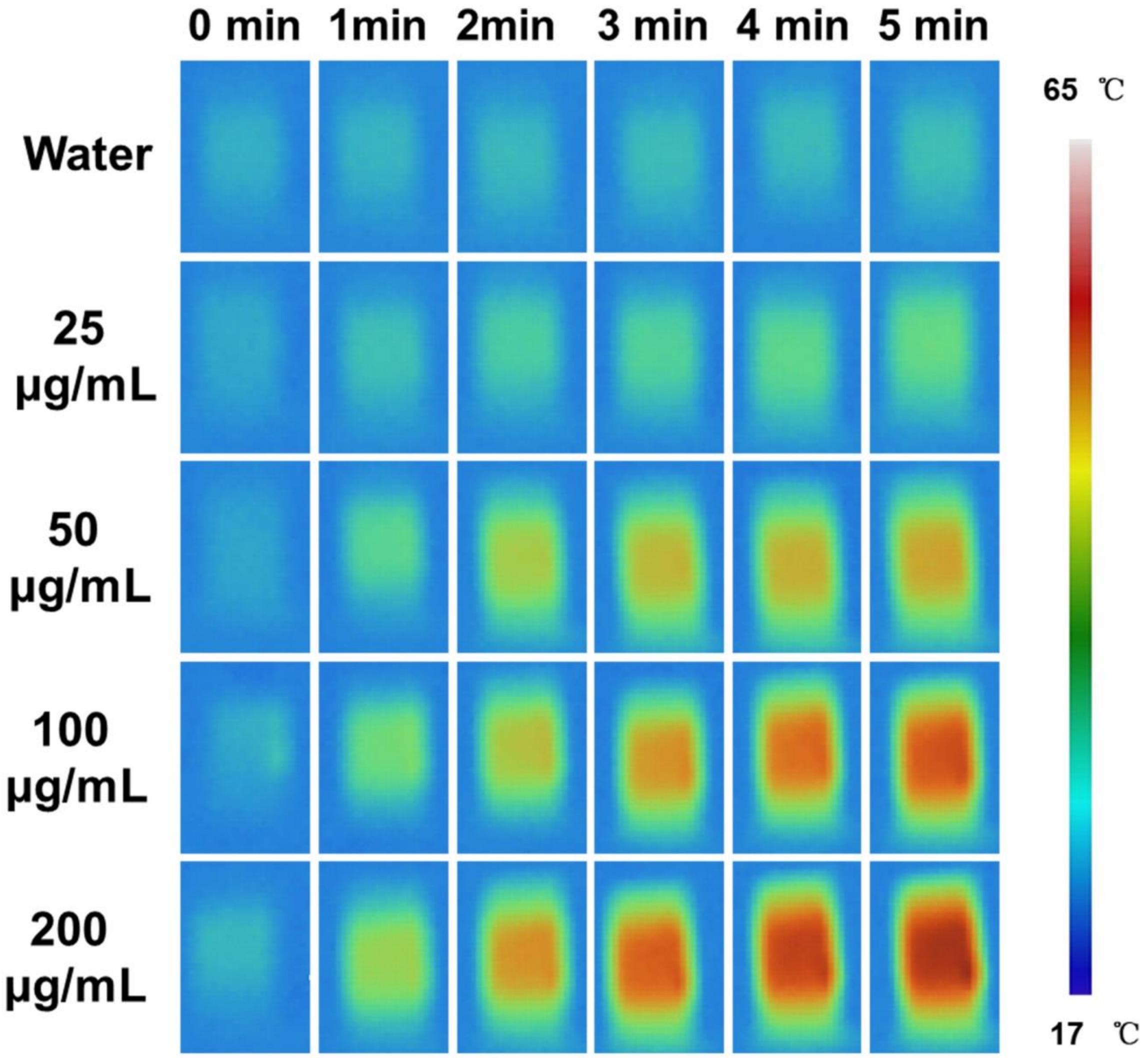


**Figure S5.** Thermal imaging of different concentrations of POM-Co (0, 25, 50, 100 and 200 µg/mL) after adding the same content of $Na_2S$ under 808 nm laser irradiation.

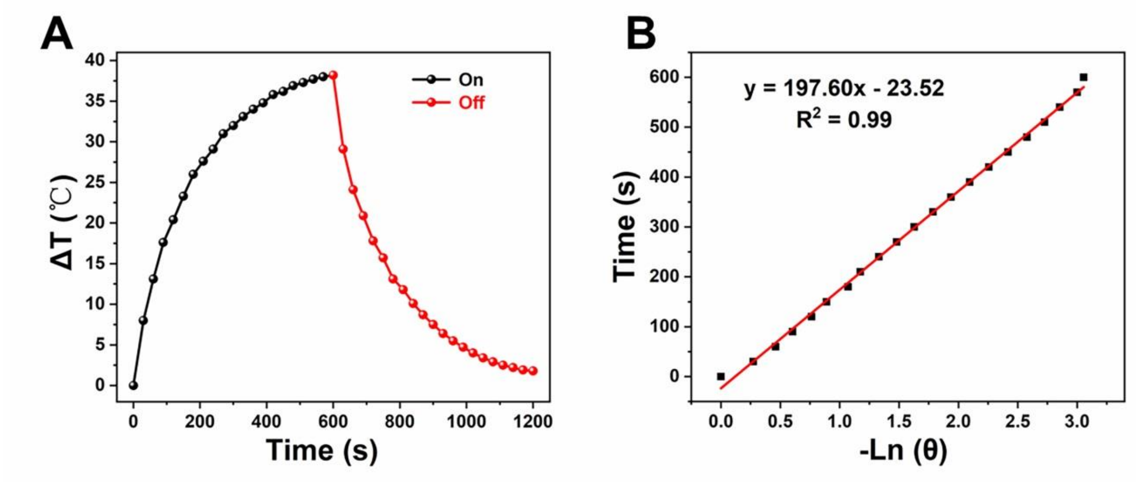


**Figure S6.** (A) Temperature variation curve of POM-CoS clusters under 808 nm laser irradiation (1.5 W/$cm^2$). (B) Fitted linear relationship between time and -Ln (θ).

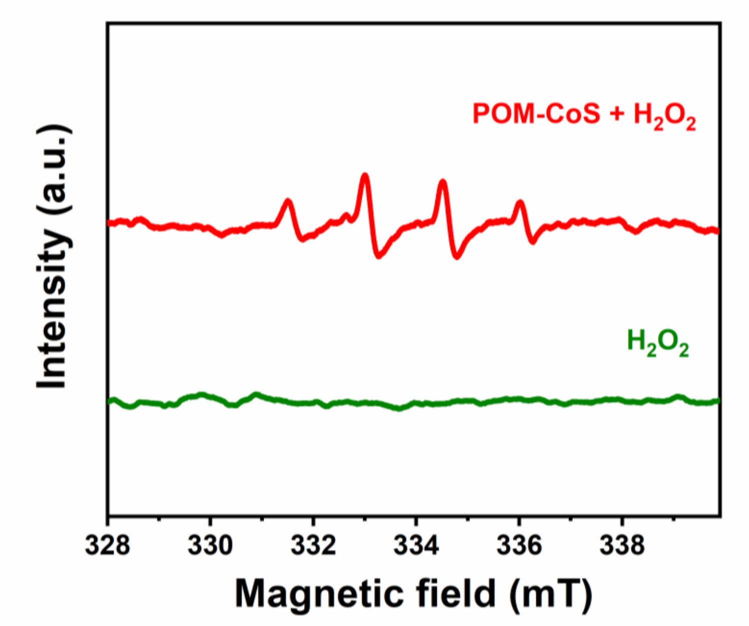


**Figure S7.** ESR spectra of •OH generation in $H_2O_2$ and POM-CoS + $H_2O_2$ samples.

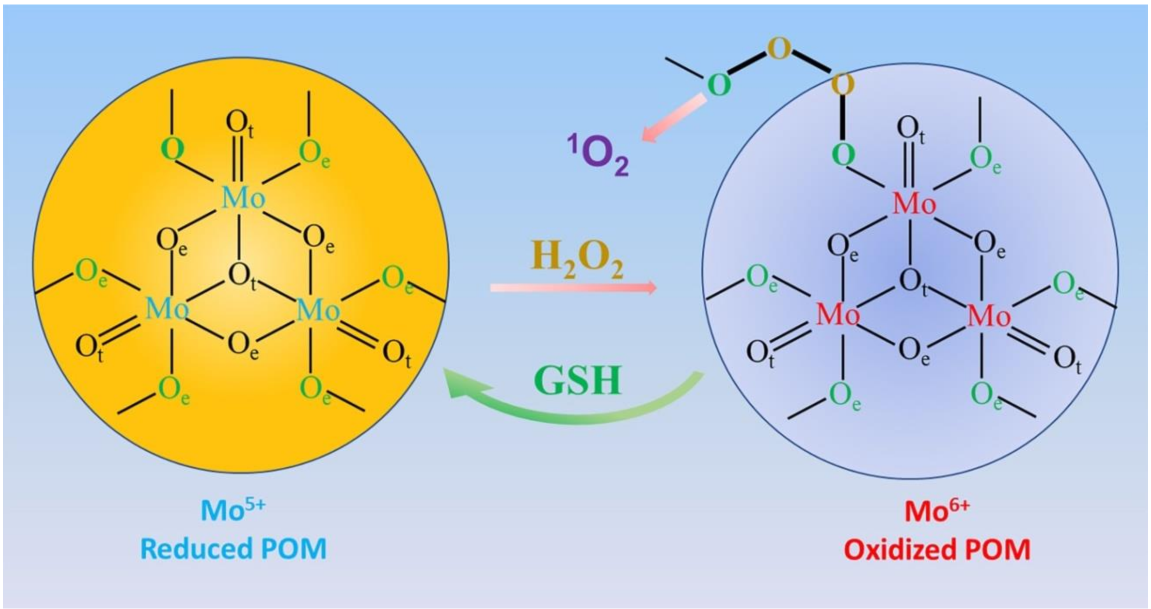


**Figure S8.** Schematic illustration of Russell mechanism for POM to generate $^1O_2$.

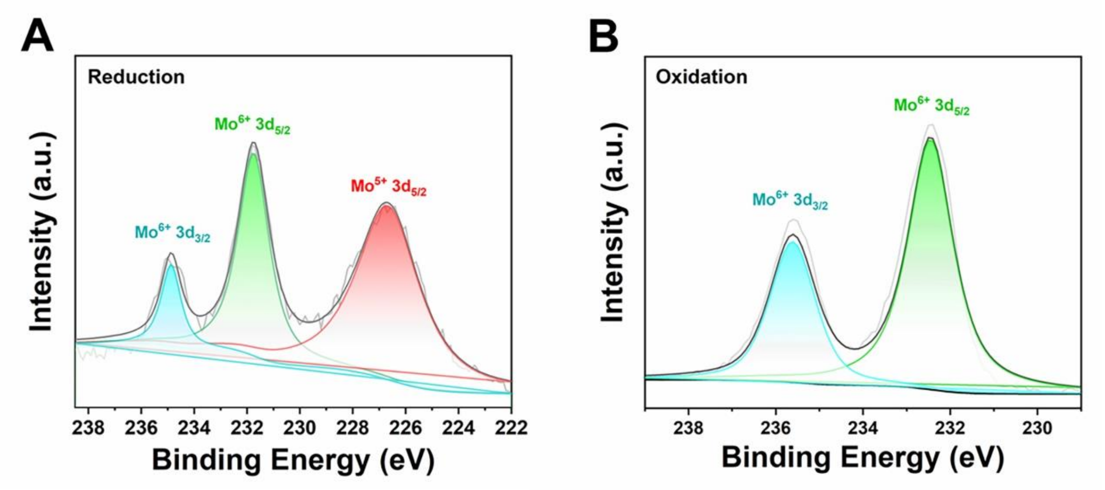


**Figure S9.** The XPS spectra of POM-CoS (Mo3d) with various treatments of (A) POM-CoS sample added with GSH, (B) POM-CoS sample sequentially added with GSH and $H_2O_2$.

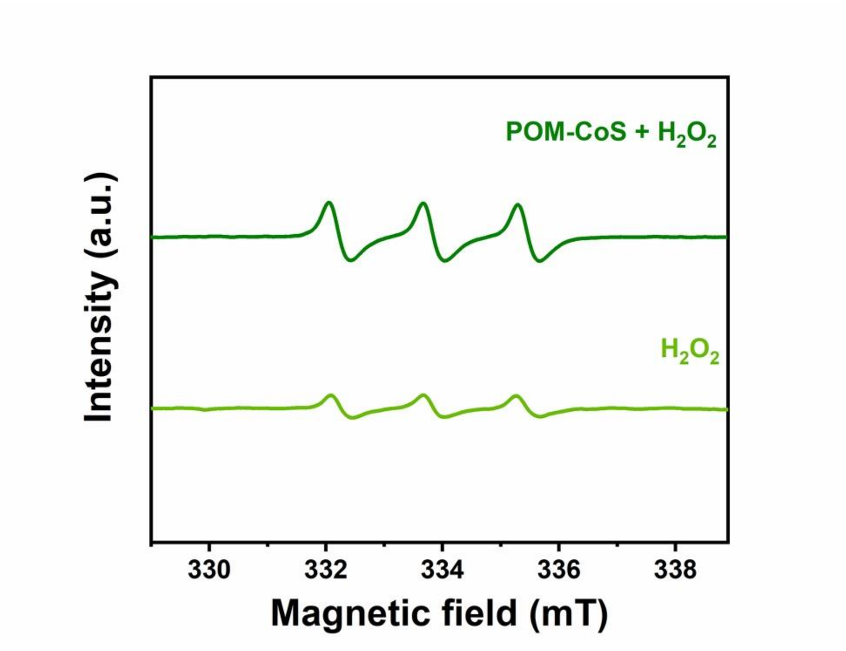


**Figure S10.** ESR spectra detection of TEMP-captured $^1O_2$.

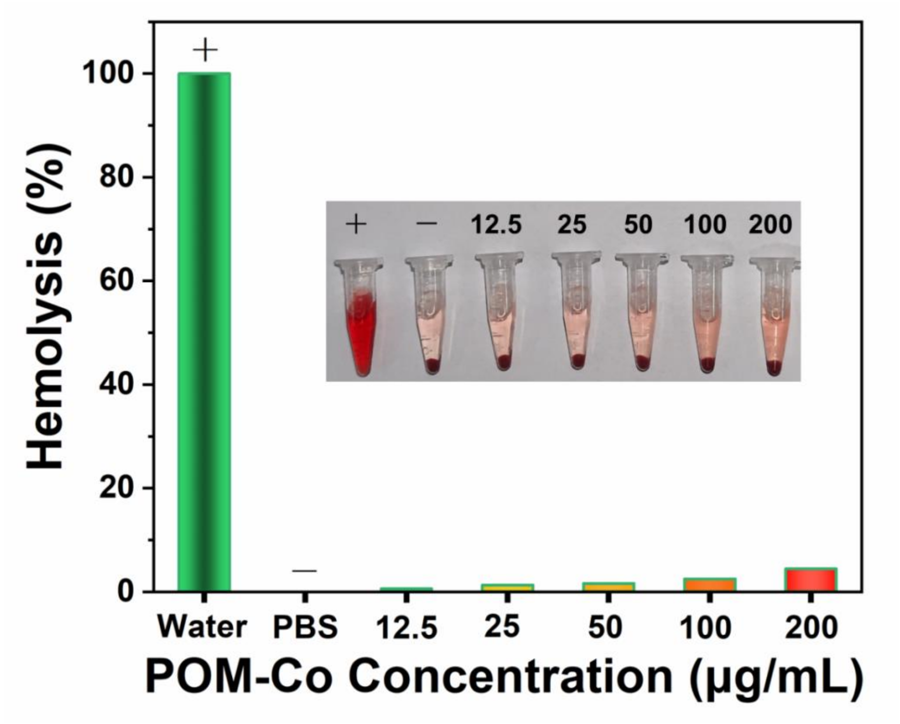


**Figure S11.** Hemolysis test of POM-Co at different concentrations.

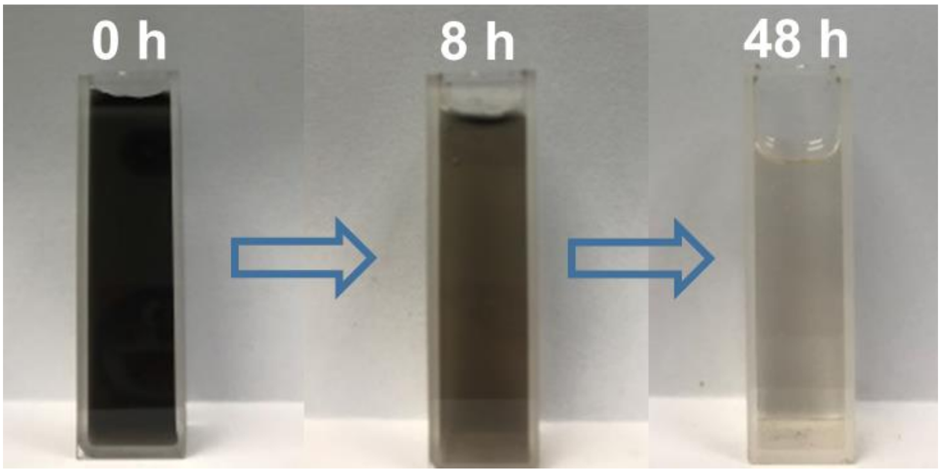


**Figure S12.** Photograph of POM-CoS dispersed in deionized water for 0, 8 and 48 h.

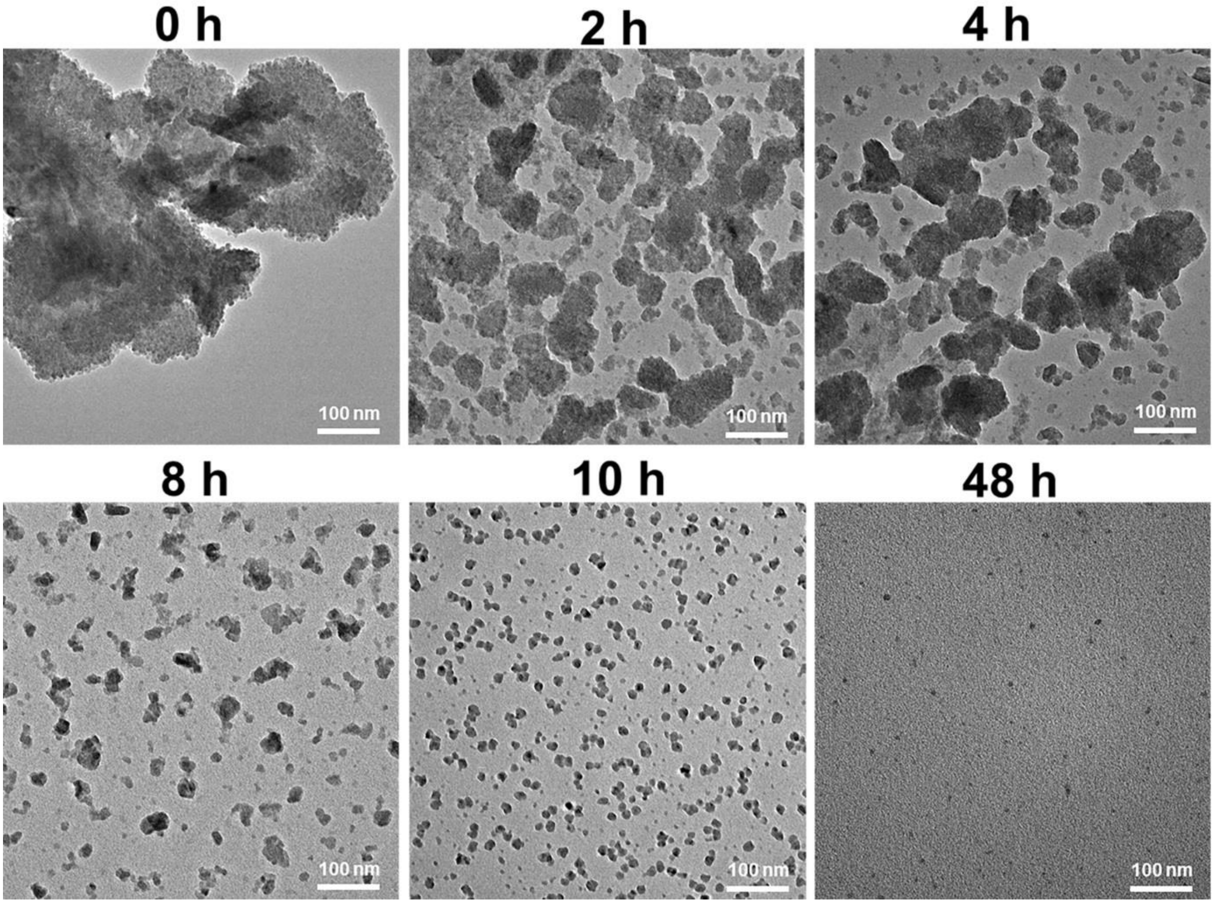


**Figure S13.** TEM images of POM-CoS nanocluster in deionized water at different time points.

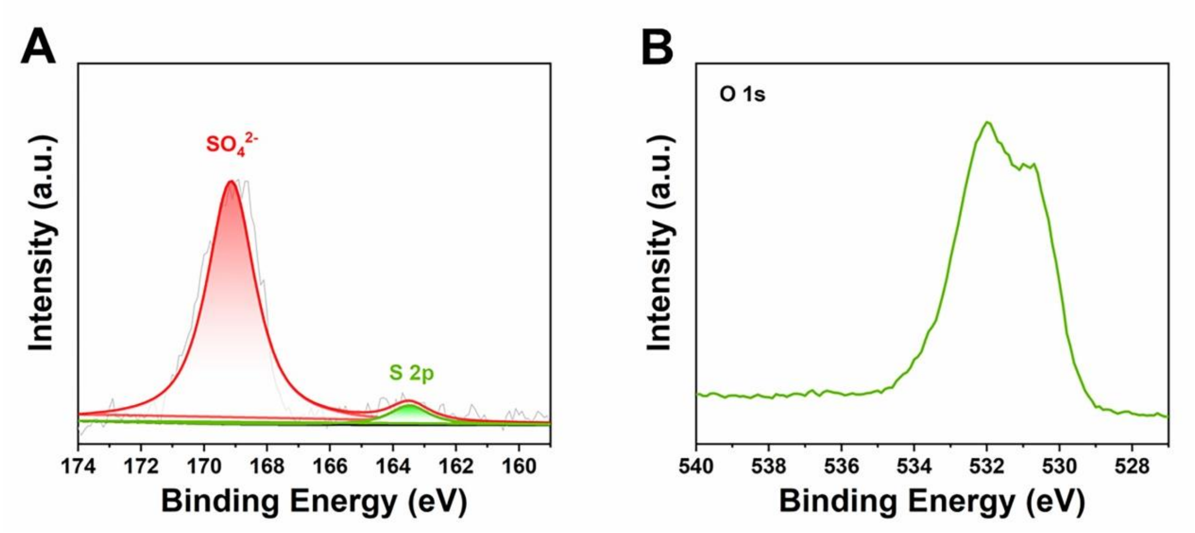


**Figure S14**. High resolution XPS spectra of (A) S and (B) O after POM-CoS biodegradation.

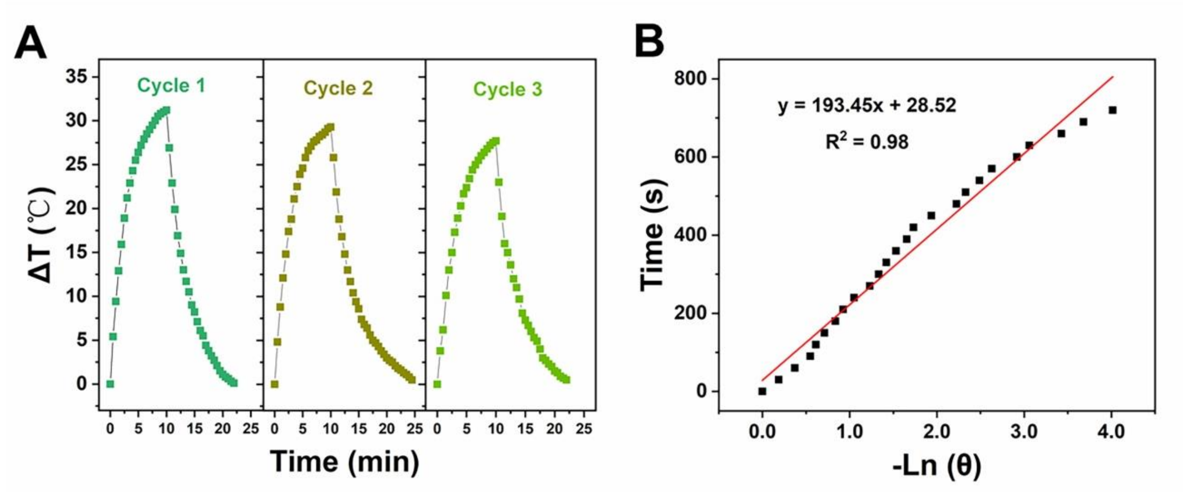


**Figure S15.** (A) Temperature curve of three-cycle activation of POM-Co clusters under 808 nm laser irradiation. (B) Fitted linear relationship between time and -Ln (θ) for cycle 3.

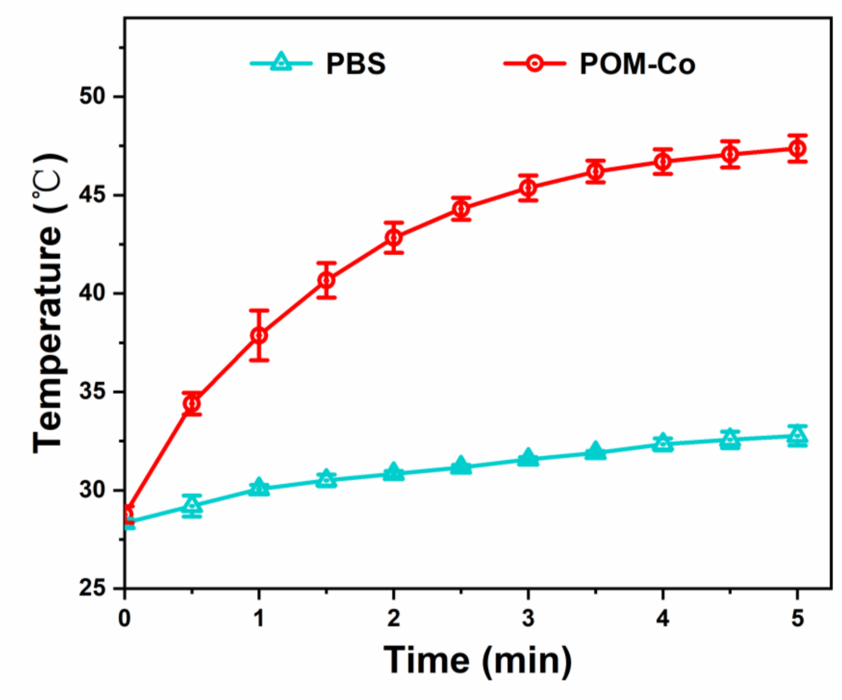


**Figure S16.** Temperature curves of tumor-bearing mice with injecting saline and POM-Co under 808 nm laser irradiation for various times.

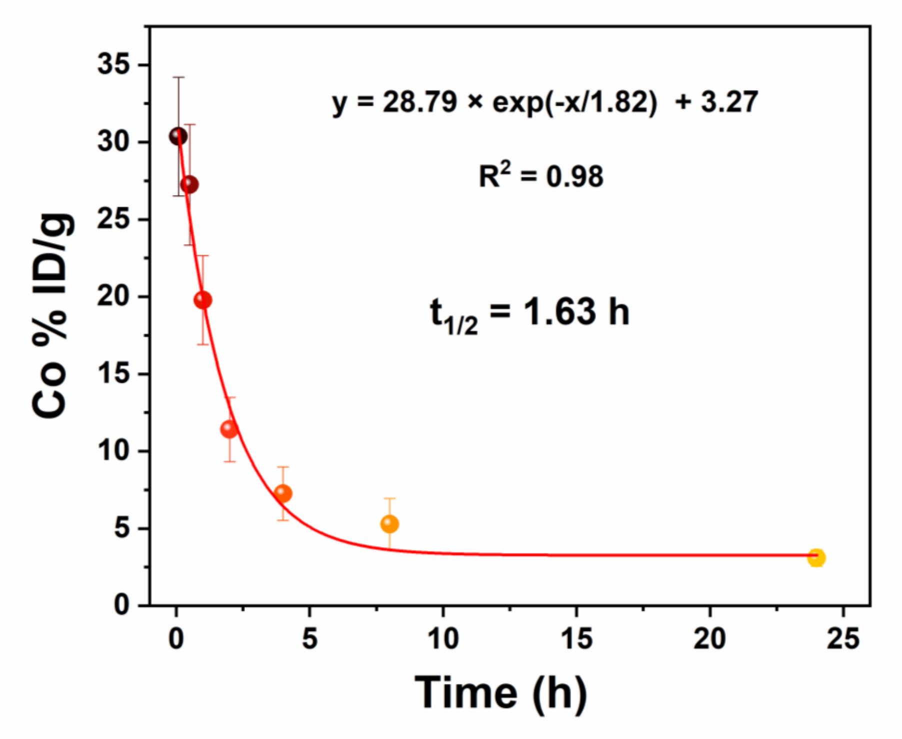


**Figure S17**. The blood circulation curve of the intravenously injected POM-Co nanocluster. The half-time ($t_{1/2}$) was calculated to be ~1.63 h.

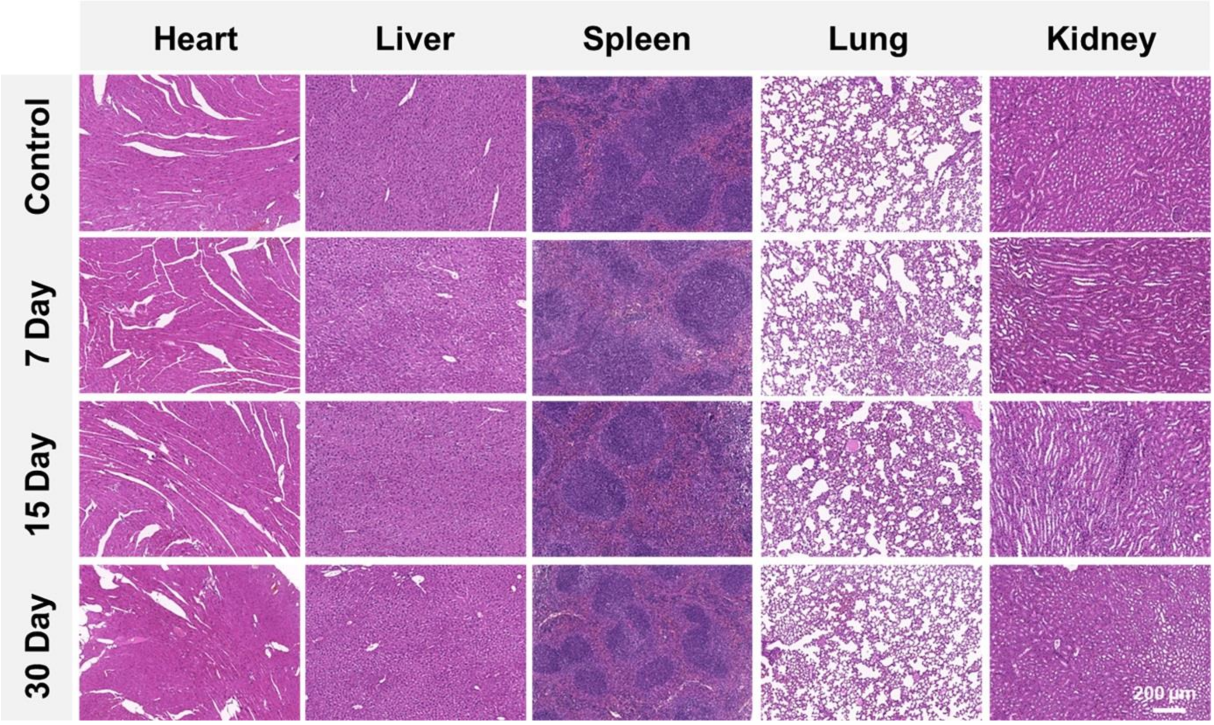


**Figure S18.** H&E stained main tissues including heart, liver, spleen, lung and kidney collected from normal mice treated without or with POM-Co clusters for 7, 15 and 30 day.

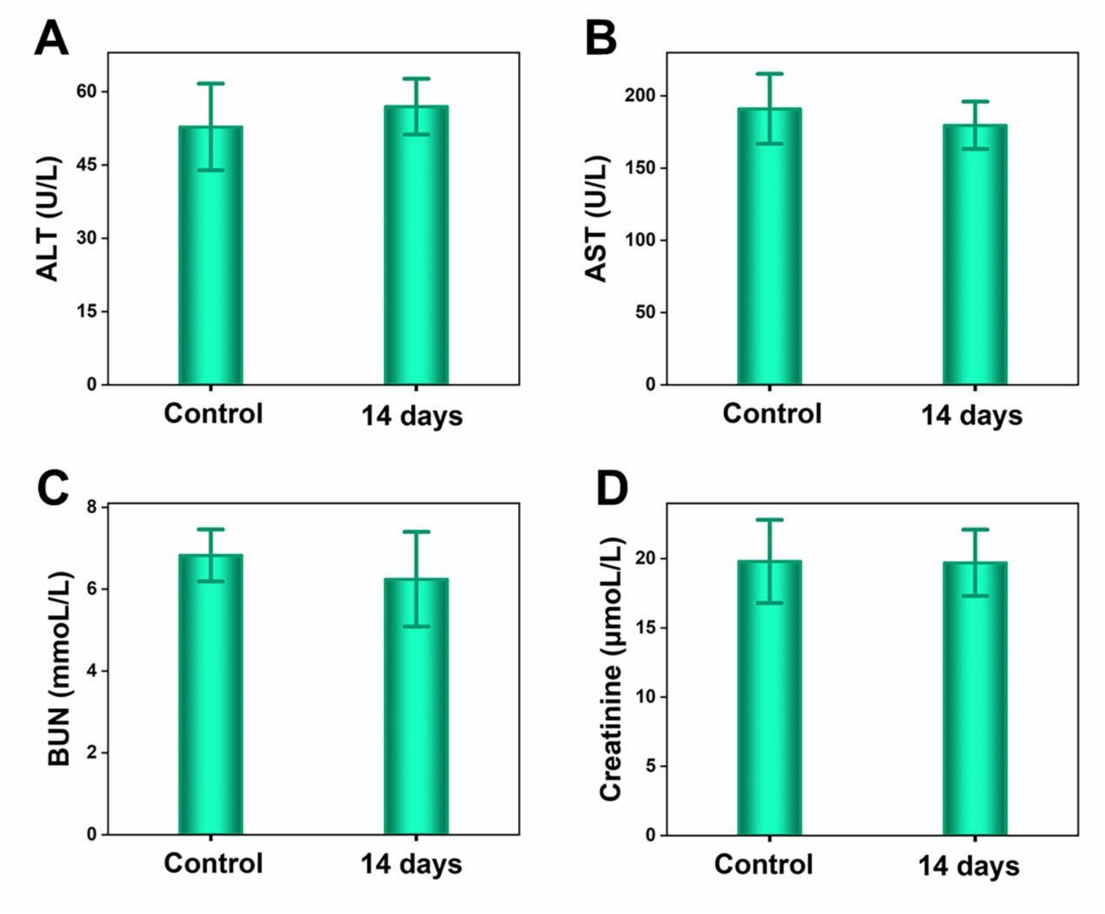


**Figure S19.** (A)-(D) *In vivo* blood biochemistry tests of the mice after intravenous injection of POM-Co nanoprobes and control mice without injection.
